# ICD-11 Posttraumatic Stress Disorder and Complex PTSD Among Hospital Medical Workers in China: Impacts of Wenchuan Earthquake Exposure, Workplaces, and Sociodemographic Factors

**DOI:** 10.3389/fpsyt.2021.735861

**Published:** 2022-01-17

**Authors:** Sijian Li, Chunlan Guo, Sunshine S. S. Chan

**Affiliations:** ^1^School of Nursing, The Hong Kong Polytechnic University, Kowloon, Hong Kong SAR, China; ^2^World Health Organization Collaborating Center for Community Health Services, The Hong Kong Polytechnic University, Kowloon, Hong Kong SAR, China

**Keywords:** posttraumatic stress disorder, complex PTSD, hospital medical workers, 2008 Wenchuan earthquake, cross-sectional study, Sichuan

## Abstract

**Background:**

Previous studies address posttraumatic stress disorder (PTSD) following disasters as a public health issue. However, few studies investigate the long-term effect of disaster exposure on PTSD among hospital medical workers (HMWs).

**Objectives:**

This study aimed to study the prevalence of ICD-11 PTSD and complex PTSD (CPTSD) among exposed and non-exposed HMWs 11 years after the Wenchuan earthquake in China, to identify the factors associated with PTSD and CPTSD scores, and to examine the factor structures of PTSD and CPTSD models.

**Methods:**

A cross-sectional study was conducted using a self-administered online questionnaire. Two thousand fifty-nine valid samples were collected from four hospitals in 2019. Descriptive statistical analysis, multivariate regression models, and confirmatory factor analysis (CFA) were performed.

**Results:**

The prevalence of PTSD and CPTSD was 0.58 and 0.34%, respectively. The unexposed group reported higher PTSD and CPTSD scores than the exposed group. The type of workplace and marital status were significantly associated with the PTSD and CPTSD scores of HMWs. The CFA results indicate that both the correlated first-order model and the correlated two-layer model were a good fit to explain the structure of PTSD and CPTSD.

**Conclusion:**

These findings suggest that few HMWs who were exposed to the Wenchuan earthquake suffered from PTSD or CPTSD 11 years following the disaster. However, psychological support was still necessary for all HMWs, especially for unmarried HMWs who were Working in smaller hospitals. Further research is required to analyze mental health status using ICD-11 PTSD and CPTSD to provide ongoing evidence to help HWMs cope effectively with the challenges of future disasters.

## Introduction

Posttraumatic stress disorder (PTSD) is one of the most common types of psychopathologies experienced after a mass traumatic incident caused by a disaster (1). The disorder involves substantial functional impairment and often coexists with other mental health conditions, such as depression, generalized anxiety disorder, and substance abuse. For these reasons, PTSD is the most frequently studied postdisaster mental health disorder (2–4). Moreover, disasters and disaster response can have long-term effects on individuals' mental health (5, 6). A cohort study of the link between hurricane experience and mental health found that adolescents at the time of the disaster had a reduced incidence of depression and PTSD up to 9 years postdisaster compared with those who were adults at the time of the disaster (7).

According to a report released by China's Ministry of Civil Affairs in 2008, the Wenchuan earthquake event of May 12, 2008, claimed 69,227 lives and injured 374,643 people, whereas 17,923 people were missing at the time of the report and 46,240,000 people were affected (8). The earthquake had a magnitude of 8.0 on the Richter scale, and its epicenter had a maximum intensity of 11.0 in Sichuan Province, southwest China. There were approximately 10,630 medical workers deployed to the affected areas to offer assistance with the rescue efforts, such as providing emergency response services, collaboration and cooperation, large-scale transportation, and the rehabilitation of those injured while responding to the earthquake (8). As the largest component of the medical care workforce, healthcare professionals often play a vital role in providing emergency medical relief when a disaster strikes (5, 9, 10). They are also vulnerable to developing PTSD (11) because they may witness mass causalities, deaths, and severe injuries, which may cause psychological shock or intense fear.

The prevalence of PTSD was relatively high directly following the Wenchuan earthquake, but its prevalence declined over time for the majority of people, according to a systematic review, which analyzed 58 original studies of PTSD prevalence in the 10 years after the earthquake among community populations (12). The prevalence of PTSD among medical rescue workers was 19.3% (13), 30.0% (11), 17.0% (14), and 1.7% (15) at 3, 6–12, and 14–17 months and 2 years after the earthquake, respectively. A longitudinal study examined adult survivors and found that the prevalence of PTSD was 58.2% at 2 months, 22.1% at 8 months, 19.8% at 14 months, 19.0% at 26 months, and 8.0% at ~44 months after the Wenchuan earthquake (16). However, studies of the long-term effects (e.g., more than 10 years) of disaster exposure on the mental health of medical rescue workers are lacking. In addition, most previous studies focusing on individuals' mental health after disasters do not include unexposed comparison groups to distinguish the effect of disaster exposure on PTSD (5).

The World Health Organization has published the 11th version of the International Classification of Diseases (ICD-11), which was approved by the World Health Assembly in 2017 (17). The initial criteria for PTSD can be traced back to 1980 in the third edition of the Diagnostic and Statistical Manual. The purpose of this updated version is to improve the utility of public health, disease-related, and clinical applications to maintain diagnostic and screening reliability and validity (18). Brewin et al. (17) further suggest that the value of ICD-11 is that it provides a brief set of systems to distinguish between two sibling disorders, PTSD and complex PTSD (CPTSD). Several studies examine the association of PTSD with disasters, such as studies of earthquake survivors in China (19, 20), Australian accident victims (21), and preadolescent children exposed to a hurricane in the United States (22). The findings of these studies indicate that the ICD-11 model has an acceptable fit for data collected from postdisaster settings.

Therefore, this study investigated ICD-11 PTSD and CPTSD among hospital medical workers (HMWs) 11 years after the Wenchuan earthquake. Specifically, the objectives were to examine (1) the prevalence of PTSD and CPTSD among earthquake-exposed and non-exposed HMWs, (2) the factors associated with their PTSD and CPTSD scores, and (3) the factor structure of PTSD and CPTSD models. The findings are significant as they demonstrate the long-term effects of PTSD and CPTSD. This helps us understand the effect of disasters on healthcare professionals' well-being and provides evidence to inform policies on the prevention of mental health disorders in the long term.

## Materials and Methods

### Design

This was a cross-sectional study. Data were collected from four hospitals from June 2019 to October 2019. These four hospitals were selected based on the following criteria: (1) geographic location as they were located either at the epicenter of the Wenchuan earthquake (Hospital D in Mianzhu, Sichuan), near the epicenter (Hospitals B and C, Chengdu, Sichuan), or a great distance from the epicenter (Hospital A, Quanzhou, Fujian) and (2) the size of the hospital, including the total number of beds and HMWs and the number of potential participants. Hospital B was the largest hospital (4,300 beds and more than 10,000 healthcare workers), and Hospital D was the smallest hospital (800 beds and approximately 1,000 healthcare workers; Table 1).

**Table 1:** General information about the four hospitals.

	**Hospital A**	**Hospital B**	**Hospital C**	**Hospital D**
Geographical location	Quanzhou, Fujian	Chengdu, Sichuan	Chengdu, Sichuan	Mianzhu, Sichuan
Hospital size	Tertiary grade A	Tertiary grade A	Tertiary grade A	Tertiary grade B
**Number of**				
Beds	2,305	4,300	1,450	800
Hospital medical workers	3,398	~10,000	~3,500	1,163
Number of participants	532 (100%)	503 (100%)	489 (100%)	535 (100%)
Medical doctor	97 (18.2%)	1 (0.2%)	22 (4.4%)	149 (27.9%)
Registered nurse	435 (81.1%)	488 (99.8%)	481 (95.6%)	386 (72.1%)

### Participants

HMWs were included in the study if they were (1) medical doctors or registered nurses working in one of the four selected hospitals and (2) willing to participate in the study and complete the online questionnaire. Those who were taking prolonged leave due to illness or maternity were excluded from the study. The sample size was large enough to ensure that parameter estimates were within the acceptable margin of error of 4% with a confidence level of 95%. Approximately 500 valid questionnaires were collected from each of the hospitals (Table 1). However, the numbers of participating medical doctors and registered nurses varied among these hospitals. The largest participation rate was from medical doctors from Hospital D (149 out of 535, 27.9%), whereas only one medical doctor (0.2%) from Hospital C completed the questionnaire.

### Measurements

The International Trauma Questionnaire (ITQ) (23) was used as a self-reported measure of ICD-11 PTSD and CPTSD. CPTSD is a disorder comprising PTSD and disturbances in self-organization (DSO) symptoms.

The ITQ measurement assesses six symptoms of PTSD distributed across the following three clusters: re-experiencing (RE with the symptoms of RE1, upsetting dreams, and RE2, reliving the events), avoidance (AV with the symptoms of AV1, internal avoidance, and AV2, external avoidance), and a sense of threat (TH with the symptoms of TH1, being on guard, and TH2, being easily startled). The six symptoms of DSO are distributed across the following three clusters: affective dysregulation (AD with the symptoms of AD1, intense reaction, and AD2, long time to calm down), negative self-concept (NSC with the symptoms of NSC1, feelings of failure, and NSC2, feelings of worthlessness), and disturbances in relationships (DR with the symptoms of DR1, feeling cut off from others, and DR2, difficulty staying close to others). All symptoms were assessed using a five-point Likert scale ranging from 0 = “not at all” to 4 = “extremely” (Table A1 in **Appendix I**).

PTSD and CPTSD prevalence: Three additional questions were used to evaluate PTSD-related functional impairment (PTSDI1, social life distress; PTSDI2, work affected; and PTSDI3, other aspects of life affected) and DSO-related functional impairment (DSOFI1, DSOFI2, and DSOFI3), also in the domains of social life, work life, and other important areas of life, respectively. Scores ≥ 2 (“moderately”) indicated the presence of a symptom. PTSD and DSO prevalence requires the presence of at least one symptom in each PTSD/DSO cluster plus the presence of functional impairment associated with these symptoms. CPTSD prevalence requires a PTSD prevalence, one symptom in each DSO cluster, and associated functional impairment. For example, if RE1 or RE2 > 2 (“moderately”), RE criteria were met; if AV1 or AV2 > 2, AV criteria were met; if TH1 or TH2 > 2, TH criteria were met; if PTSDI1, PTSDI2, or PTSDI3 > 2, PTSDI criteria were met; and if RE, AV, TH, and PTSDI criteria were all met, a diagnosis of PTSD was made. The same criteria were used for CPTSD prevalence. PTSD and CPTSD were assessed using a self-reported measure rather than a clinician-administered assessment. For this reason, the term “probable” is used when referring to estimating the prevalence of PTSD and CPTSD.

Wenchuan earthquake exposure: The participants were divided into the following three groups according to their level of exposure to the Wenchuan earthquake: unexposed HMWs, earthquake survivors (who were adolescents in 2008 and were exposed to the earthquake as survivors), and earthquake relief workers (who were adults in 2008 and were exposed to the earthquake as HMWs).

Workplaces: Four hospitals participated in the survey (Hospitals A, B, C, and D). They had different geographical locations and different numbers of HMWs and beds (Table 1).

Sociodemographic factors: The sociodemographic data that were collected included gender (male/female), educational level (college or below/university or above), marital status (unmarried/married), profession (medical doctor/registered nurse), age, and length of service in years.

### Data Analysis

First, a descriptive analysis was performed to understand the characteristics of the sample and determine the prevalence of PTSD and CPTSD. Second, the PTSD and DSO scores were calculated for different groups of HMWs. Multivariate regression models were established to examine the effect of Wenchuan earthquake exposure, the type of workplace, and sociodemographic factors on the PTSD and DSO scores of the HMWs. All analyses were performed using SPSS software, version 25.0 (IBM, Armonk, NY, the United States) with the threshold for statistical significance at a two-tailed α value of 0.05. The beta coefficient (β), the associated two-tailed *p*-values (*p*), and 95% confidence intervals (CIs) were reported for each independent variable in the regression models. An analysis of variance test was performed, and *p*- and *R*^2^-values were reported to evaluate the performance of each regression model.

Third, confirmatory factor analysis (CFA) was performed using Mplus 7.11 (Muthén and Muthén, Los Angeles, CA, the United State) to examine the structure of PTSD and CPTSD in HMWs. Four different models were established for CFA based on data from multiple trials (24–26). Model 1 was a one-layer, one-factor model in which the latent factor of CPTSD was constructed by the 12 ITQ symptoms. Model 2 was a correlated six-factor model in which each of the clusters of PTSD (RE, AV, and TH) and DSO (AD, NSC, and DR) was a latent factor. The correlation between the six latent factors was tested in Model 2. Model 3 was a two-layer model in which CPTSD was constructed using the six latent factors in Model 2. Model 4 was a correlated two-layer model in which PTSD accounted for the covariation between the latent factors of RE, AV, and TH, whereas DSO accounted for the covariation between the latent factors of AD, NSC, and DR. The maximum likelihood estimation was used, and the STDYX standardization of estimate together with its standard errors (SE) were reported for each path in the CFA.

The comparative fit index (CFI), root mean square error of approximation (RMSEA) with 95% CI, and Tucker–Lewis index (TLI) were used to assess the fit of the hypothetical model. Cutoff values of 0.95 for CFI and TLI (a higher value is better) and 0.06 for RMSEA (a lower value is better) (27, 28) were used to determine the goodness of fit. To identify the model with the optimal fit, changes in the CFI (ΔCFI), TLI (ΔTLI), and RMSEA (ΔRMSEA) values were assessed. ΔCFI ≥ 0.01, ΔTLI ≥ 0.01, and ΔRMSEA ≥ 0.015 were considered to be evidence of a meaningful difference in the fit of the respective models (29).

## Results

### Demographic Characteristics and Prevalence of ICD-11 PTSD and CPTSD

Of the 2,059 HMWs in the study, 9.9% experienced the Wenchuan earthquake as survivors (204 out of 2,059), 15.4% experienced the earthquake as disaster relief workers (317 out of 2,059), and 74.7% did not experience the earthquake (1,538 out of 2,059; Table 2). There were more female than male HMWs in the total sample (1,791 vs. 268). More participants had a college education level or below (including diploma and subdegree) than a university educational level or above (1,193 vs. 866). More of the participants were married than unmarried (1,355 vs. 704). More than 80% of the participants were registered nurses (1,790 of 2,059, 86.9%). The mean age of the participants was 31.4 with a standard deviation (SD) of 7.1, whereas the mean length of service as an HMW was 9.6 years with an SD of 7.6 years.

**Table 2:** Rates of PTSD and CPTSD in HMWs 11 years after the Wenchuan Earthquake.

	**Total** **(*N* = 2,059)** ***N* (%)**	**ICD-11 PTSD and CPTSD**		
		**Normal** **(*N* = 2,040)** ***N* (%)**	**PTSD prevalence** **(*N* = 12)** ***N* (%)**	**CPTSD prevalence** **(*N* = 7)** ***N* (%)**
**Wenchuan earthquake exposure**				
Unexposed HMWs	1,538 (100%)	1,521 (98.9%)	12 (0.8%)	5 (0.3%)
Earthquake survivors	204 (100%)	203 (99.5%)	0 (0.0%)	1 (0.5%)
Earthquake relief workers	317 (100%)	316 (99.7%)	0 (0.0%)	1 (0.3%)
**Workplace**				
Hospital A	532 (100%)	520 (97.7%)	7 (1.3%)	5 (0.9%)
Hospital B	503 (100%)	501 (99.6%)	2 (0.4%)	0 (0.0%)
Hospital C	489 (100%)	487 (99.6%)	1 (0.2%)	1 (0.2%)
Hospital D	535 (100%)	532 (99.4%)	2 (0.4%)	1 (0.2%)
**Gender**				
Male	268 (100%)	264 (98.5%)	3 (1.1%)	1 (0.4%)
Female	1,791 (100%)	1,776 (99.2%)	9 (0.5%)	6 (0.3%)
**Education**				
College or below	1,193 (100%)	1,181 (99.0%)	6 (0.5%)	6 (0.5%)
University or above	866 (100%)	859 (99.2%)	6 (0.7%)	1 (0.1%)
**Marital status**				
Unmarried	704 (100%)	697 (99.0%)	5 (0.7%)	2 (0.3%)
Married	1,355 (100%)	1,343 (99.1%)	7 (0.5%)	5 (0.4%)
**Profession**				
Medical doctor	269 (100%)	265 (98.5%)	4 (1.5%)	0 (0.0%)
Registered nurse	1,790 (100%)	1,775 (99.2%)	8 (0.4%)	7 (0.4%)
**Age**	31.4 (7.1)	31.4 (9.6)	31.7 (10.7)	28.1 (4.9)
Length of service in years	9.6 (7.6)	9.6 (7.6)	10.5 (10.7)	6.4 (4.6)

The prevalence of PTSD and CPTSD was 0.58% (12 of 2,059) and 0.34% (7 of 2,059), respectively. All participants with PTSD (100%, 12 of 12) and most of those with CPTSD (71.4%, 5 of 7) had not been exposed to the Wenchuan earthquake. Approximately equal numbers of participants were recruited from the four hospitals. However, Hospital A reported the highest prevalence of PTSD (1.3%, 7 of 521) and CPTSD (0.9%, 5 of 521). Moreover, male participants reported a higher prevalence of both PTSD (1.1 vs. 0.5%) and CPTSD (0.4 vs. 0.3%) than their female HMW counterparts. The participants with an education at the university level or above reported a higher prevalence of PTSD (0.7 vs. 0.5%) and a lower prevalence of CPTSD (0.1 vs. 0.5%) than their counterparts with lower educational levels. A higher prevalence of PTSD (0.7 vs. 0.5%) but a lower prevalence of CPTSD (0.3 vs. 0.4%), was reported by unmarried participants compared with married participants. Medical doctors reported a much higher prevalence of PTSD (1.5 vs. 0.4%) than registered nurses, whereas all cases of CPTSD were found in registered nurses. The mean age was lower and the length of service as an HMW was shorter in those with CPTSD than in those with PTSD and those without CPTSD or PTSD.

### Dimensional Scores of PTSD and DSO and Their Determinants

Participants, who were probable with CPTSD, reported the highest PTSD (mean = 26.86, SD = 3.58) and DSO (mean = 27.29, SD = 5.82) scores, followed by those with PTSD (Table 3). Participants with different types of Wenchuan earthquake exposure reported significantly different of PTSD [*F*_(2)_ = 17.281, *p* < 0.001] and DSO [*F*_(2)_ = 20.547, *p* < 0.001] scores. Unexposed HMWs reported the highest PTSD (mean = 5.53, SD = 5.58) and DSO (mean = 5.73, SD = 6.06) scores.

**Table 3:** Dimensional PTSD and DSO scores of HMWs.

	**PTSD score**	**DSO score**
ICD-11-defined PTSD and CPTSD prevalence	F(2)= 154.896, *p* < 0.001	F(2)= 89.685, *p* < 0.001
Normal	4.95 (4.98)	5.11 (5.54)
PTSD	24.00 (2.49)	18.33 (4.70)
CPTSD	26.86 (3.58)	27.29 (5.82)
Wenchuan earthquake exposure	F(2)= 17.281, *p* < 0.001	F(2)= 20.547, *p* < 0.001
Unexposed HMWs	5.53 (5.58)	5.73 (6.06)
Earthquake survivors	3.74 (4.12)	3.93 (4.54)
Earthquake relief workers	4.12 (4.41)	3.85 (4.51)
Workplace	F(3)= 11.985, *p* < 0.001	F(3)= 12.973, *p* < 0.001
Hospital A	6.24 (6.08)	6.49 (6.55)
Hospital B	4.35 (4.74)	4.68 (5.28)
Hospital C	5.01 (5.19)	5.36 (5.79)
Hospital D	4.89 (4.99)	4.52 (5.11)
Gender	F(1)= 2.741, *p* = 0.098	F(1)= 3.221, *p* = 0.073
Male	5.64 (5.68)	5.85 (6.39)
Female	5.06 (5.27)	5.18 (5.66)
Education	F(1)= 0.192, *p* = 0.662	F(1)= 2.360, *p* = 0.125
College or below	5.09 (5.36)	5.10(5.68)
University or above	5.20 (5.29)	5.49(5.87)
Marital status	F(1)= 0.593, *p* = 0.441	F(1)= 7.453, *p* < 0.01
Unmarried	5.26 (5.44)	5.75 (6.34)
Married	5.07 (5.27)	5.02 (5.43)
Profession	F(1)= 3.647, *p* = 0.056	F(1)= 1.464, *p* = 0.227
Medical doctor	5.71 (5.69)	5.66 (6.37)
Registered nurse	5.05 (5.27)	5.21 (5.67)

Participants from different hospitals showed significantly different PTSD [*F*_(3)_ = 11.985, *p* < 0.001] and DSO [*F*_(3)_ = 12.973, *p* < 0.001] scores. Participants from Hospital A reported the highest PTSD (mean = 6.24, SD = 6.08) and DSO (mean = 6.49, SD = 6.55) scores, followed by those from Hospital C (PTSD: mean = 5.01, SD = 5.19; DSO: mean = 5.36, SD = 5.79). Moreover, participants with different marital statuses had significantly different DSO scores [*F*_(1)_ = 7.453, *p* < 0.01].

According to the multivariate regression models (Table 4), experiencing the Wenchuan earthquake as a survivor had a significant negative effect on PTSD (β = −1.326, 95% CI: −2.160, −0.493) and DSO (β = −1.153, 95% CI: −2.053, −0.253) scores. Being a relief worker after the earthquake also had a significant negative effect on PTSD (β = −1.188, 95% CI: −1.883, −0.492) and DSO (β = −1.481, 95% CI: −2.232, −0.729) scores.

**Table 4:** Multivariate regression analysis of the effects of disaster exposure and sociodemographic factors on the dimensional scoring of PTSD and DSO.

		**PTSD score**			**DSO Score**		
		**β**	**95% CI**	***P*-value**	**β**	**95% CI**	***P*-value**
Wenchuan earthquake exposure (Ref. unexposed HMWs)	Earthquake survivors	−1.326	−2.160, −0.493	<0.001	−1.153	−2.053, −0.253	<0.05
	Earthquake relief workers	−1.188	−1.883, −0.492	<0.01	−1.481	−2.232, −0.729	<0.001
Workplace (Ref. Hospital A)	Hospital B	−1.521	−2.211, −0.831	<0.001	−1.335	−2.080, −0.590	<0.001
	Hospital C	−0.985	−1.669, −0.300	<0.01	–0.708	–1.447, –0.031	0.060
	Hospital D	−0.859	−1.556, −0.163	<0.05	−1.279	−2.032, −0.527	<0.01
Gender (Ref. male)	Female	–0.315	–1.156, 0.526	0.463	–0.485	–1.394, 0.424	0.295
Education (Ref. College or below)	University or above	–0.006	–0.518, 0.507	0.983	0.296	–0.258, 0.849	0.295
Marital status (Ref. unmarried)	Married	–0.133	–0.673, 0.406	0.628	−0.697	−1.280, −0.115	<0.05
Profession (Ref. medical doctor)	Registered nurse	–0.111	–1.039, 0.817	0.814	0.198	–0.805, 1.200	0.699
Age	Years	0.001	–0.103, 0.104	0.995	0.057	–0.055, 0.169	0.320
Length of service	Years	0.030	–0.062, 0.123	0.524	–0.013	–0.113, 0.087	0.803
Model performance		F(11)= 5.451, *p* < 0.001 R^2^ = 0.028			F(11)= 6.937, *p* < 0.001 R^2^ = 0.033		

*β coefficients, p-values, and 95% CIs are reported for each factor*.

The type of workplace also had a significant effect on the HMWs' PTSD and DSO scores. Specifically, working in Hospital D (located at the epicenter) was associated with a decrease in the PTSD score of 0.859 SDs (β = −0.859, 95% CI: −1.556, −0.163) and a decrease in the DSO score of 1.279 SDs (β = −1.279, 95% CI: −2.032, −0.527) while holding Wenchuan earthquake exposure and sociodemographic factors constant. Similarly, working in Hospital B (close to the epicenter) was associated with a decrease in the PTSD score of 1.521 SDs (β = −1.521, 95% CI: −2.211, −0.831) and a decrease in the DSO score of 1.335 SDs (β = −1.335, 95% CI: −2.080, −0.590) while holding Wenchuan earthquake exposure and sociodemographic factors constant.

In addition, being married was associated with a decrease in the DSO score of 0.697 SDs (β = −0.697, 95% CI: −1.280, −0.115) while holding Wenchuan earthquake exposure, type of workplace, and other sociodemographic factors constant. Finally, none of the associations of HMWs' PTSD or DSO scores with gender, educational level, profession, age, and length of service was statistically significant (Table 4).

### The Structure of ICD-11 PTSD and CPTSD in HMWs

**Figure 1:**
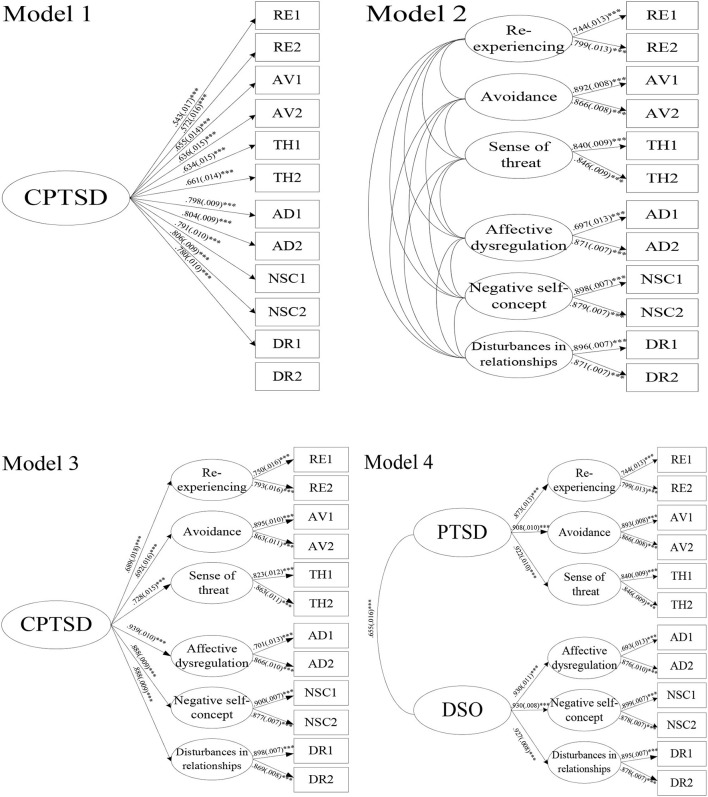
Factor models of ICD-11 PTSD and CPTSD tested using confirmatory factor analysis. The STDYX standardization of estimate and SE for Model 2: RE with AV, 0.802(0.007)***; RE with TH, 0.795(0.016)***; RE with AD, 0.596(0.022)***; RE with NSC, 0.505(0.022)***; RE with DR, 0.508(0.022)***; AV with TH, 0.837(0.012)***; AV with AD, 0.605(0.019)***; AV with NSC, 0.508(0.020)***; AV with DR, 0.525(0.019)***; TH with AD, 0.636(0.019)***; TH with NSC, 0.558(0.020)***; TH with DR, 0.525(0.019)***; AD with NSC, 0.861(0.012)***; AD with DR, 0.855(0.012)***; NSC with DR, 0.875(0.009)***.

The CFA results are presented in Table 5; Figure 1. The chi-square statistics were significant for all four models. However, Models 1 and 3 were rejected because of their poor fit. Models 2 and 4 had acceptable fits based on the CFI, TLI, RMSEA, and standardized root mean squared residual (SRMR) values. Model 2 demonstrated the highest CFI and TLI values and the lowest RMSEA and SRMR values. However, the ΔCFI and ΔTLI values between Models 2 and 4 were lower than 0.01, and the ΔRMSEA value was lower than 0.015. Thus, the difference in fit between Models 2 and 4 was not significant. Therefore, both Models 2 and 4 had a good fit to examine the constructs of PTSD and CPTSD in HMWs.

**Table 5:** Fit of CFA models of ICD-11 PTSD and CPTSD.

**Model**	**χ2**	**df**	***P-*value**	**CFI**	**TLI**	**RMSEA (95% CI)**	**SRMR**
Model 1	4638.142	54	<0.001	0.724	0.663	0.203 (0.198-0.208)	0.108
Model 2	215.328	39	<0.001	0.989	0.982	0.047 (0.041-0.053)	0.015
Model 3	1699.787	48	<0.001	0.901	0.863	0.129 (0.124-0.135)	0.092
Model 4	277.450	47	<0.001	0.986	0.981	0.049 (0.044-0.055)	0.026

*N = 2,059; estimator, maximum likelihood (ML); χ^2^, chi-square goodness-of-fit statistic; df, degrees of freedom; CFI, comparative fit index; TLI, Tucker–Lewis index; RMSEA (95% CI), root-mean-square error of approximation with 95% confidence interval; SRMR, standardized root mean squared residual*.

All of the factors loaded in Models 2 and 4 were significant (*p* < 0.001) with coefficients ranging from 0.693 (SE = 0.013, AD1 loaded on affective dysregulation) in Model 4 to 0.930 (SE = 0.008, negative self-concept loaded on DSO; SE = 0.011, affective dysregulation loaded on DSO) in Model 4. All correlations between the six latent factors were significant with coefficients ranging from 0.508 (RE with DR, SE = 0.22; and AV with NSC, SE = 0.20) to 0.875 (NSC with DR, SE = 0.009) in Model 2. In Model 4, the latent factors of RE, AV, and TH constructed the second-layer latent factor of PTSD, while AD, NSC, and DR constructed DSO. PTSD was significantly correlated with DSO with a medium-sized effect (β = 0.655, SE = 0.016).

## Discussion

### PTSD and CPTSD in HMWs After the Wenchuan Earthquake

The prevalence rate of PTSD and CPTSD in HMWs was low (0.58 and 0.34%, respectively) 11 years after the Wenchuan earthquake. Moreover, none of the participants who reported Wenchuan earthquake exposure were probable for PTSD. After such a long time, many cases of PTSD may have been treated or spontaneously resolved. In some cases, the rescuers who may have had PTSD had left the HMW labor market and could not be recruited for this study. Moreover, in general, the prevalence of PTSD is lower after natural disasters than after manmade disasters (30). For example, the prevalence of PTSD ranged from 7 to 24% (mean 18%) in medical responders in the first 1 or 2 years following the 2004 tsunami in Asia (31), the 2010 Yushu earthquake (32), and the 2011 Great East Japan Earthquake (33). However, after manmade disasters, the prevalence of PTSD has a wide range from 1 to 90% (34–36). The low PTSD prevalence (1%) reported for nurses and doctors during violent events in Judea and Samaria (36) was mostly related to a highly restricted selection process and more comprehensive training. A high prevalence of PTSD was identified during violent wars in 2012 (35, 70%) and Israel-Gaza in 2014 (34, 90%). As these wars continued for a long duration, they may have caused daily traumatic events. Therefore, all HMWs are urged to be equipped with competency-driven, high-quality disaster education and training to enhance their knowledge and skills to meet the global challenge of disasters (9, 10, 37).

### The Factors Associated With HMWs' PTSD and CPTSD Scores

The factors that had a significant and positive association with HMWs' PTSD and CPTSD scores were not being exposed to the earthquake and working in a hospital that was distant from the epicenter (i.e., Hospital A). These findings confirm that geographical location has a significant role in an individual's adaptation following a disaster as previously reported (38). Another possible explanation for these findings is that we only examined the effect of Wenchuan earthquake exposure on PTSD and did not evaluate the effects of other natural disasters (e.g., floods) on HMWs' mental health. Hospital A was distant from the epicenter of the Wenchuan earthquake but is located in a coastal area where typhoons, rainstorms, and high tides have become common in recent years (39). For example, in 2016, Typhoon Megi, which landed in Fujian, resulted in 91 deaths, 16 missing persons, and the collapse of 10,203 houses (40). HMWs who were working in Hospital A may have been deployed to the affected area of Fujian and exposed to traumatic incidents during flood relief work, which may have contributed to the development of PTSD in these HMWs (41).

In addition to earthquake exposure and the type of workplace, sociodemographic factors were also associated with HMWs' PTSD and CPTSD scores. An unmarried status was positively associated with PTSD and CPTSD scores, which is consistent with the findings of previous studies (41). This may be due to a lack of family and social support.

### The Structure of ICD-11 PTSD and CPTSD in HMWs

The results from the CFA indicate that both the correlated first-order model (Model 2) and the correlated two-layer model (Model 4) had a good fit to explain the structure of PTSD and DSO. The correlated first-order model, which was constructed with three latent variables (RE, AV, and TH) representing PTSD and three latent variables (AD, NSC, and DR) representing DSO, was the model with the best fit. The correlated two-layer model included two second-order latent variables, PTSD and DSO, to explain the covariation among the six first-order factors. These results are consistent with previous studies showing that these two models are generally the best-fitting models (42–44).

### Limitations and Further Research

First, this study was limited by its sampling methodology and the unbalanced ratio between medical doctors (18.2%) and registered nurses (81.1%) from the four selected hospitals. This may limit the generalizability of the finding that being a medical doctor predicted the development of PTSD and CPTSD. Second, this study only assessed HMWs' exposure to the Wenchuan earthquake, which occurred 11 years prior to the survey. There may have been other traumatic events during that period that caused PTSD or CPTSD. Therefore, a life event checklist for the past 11 years may be warranted to evaluate the effect of other traumatic events during this period. Third, we used an online self-reported survey, and we did not conduct interviews to make the diagnoses. Finally, this was a cross-sectional study with a non-random sampling strategy. With the recent increase in the number of disasters globally, a long-term longitudinal study is needed to serve as a guide to advocating for the protection of the mental health of healthcare professionals working in hospitals of different sizes.

## Conclusions

The findings of this study suggest that HMWs who were exposed to the Wenchuan earthquake rarely reported PTSD or CPTSD 11 years following the disaster. However, psychological support is still necessary for all HMWs, especially for single HMWs working in smaller hospitals. Further research is required to evaluate the mental health status of HMWs using the ICD-11 criteria for PTSD and CPTSD to provide ongoing evidence to help HMWs cope effectively with the challenges of future disasters.

## Data Availability Statement

The raw data supporting the conclusions of this article will be made available by the authors, without undue reservation.

## Ethics Statement

The studies involving human participants were reviewed and approved by the Human Subjects Ethics Sub-Committee in the Hong Kong Polytechnic University (Ref.HSEARS20190416035). The patients/participants provided their written informed consent to participate in this study.

## Author Contributions

SL and SC designed and performed data collection. CG, SL, and SC analyzed the data. CG and SL drafted the manuscript. All authors contributed to the article and approved the article submission.

## Funding

This study was supported by Dean's Reserve Grants (FHSS and FENG) from the Hong Kong Polytechnic University (Project ZZHH).

## Conflict of Interest

The authors declare that the research was conducted in the absence of any commercial or financial relationships that could be construed as a potential conflict of interest.

## Publisher's Note

All claims expressed in this article are solely those of the authors and do not necessarily represent those of their affiliated organizations, or those of the publisher, the editors and the reviewers. Any product that may be evaluated in this article, or claim that may be made by its manufacturer, is not guaranteed or endorsed by the publisher.

## Appendix I

**TABLE A1:** Descriptive analysis of items of ICD-11 posttraumatic stress disorder (PTSD) and Complex PTSD.

		**Items**	**0** ***N*(%)**	**1** ***N*(%)**	**2** ***N*(%)**	**3** ***N*(%)**	**4** ***N*(%)**	**Mean** **(S.D.)**
Post-traumatic stress disorder (PTSD)	Re-experiencing	RE1. Having upsetting dreams that replay part of the experience or are clearly related to the experience	892 (43.3%)	957 (46.5%)	143 (6.9%)	61 (3.0%)	6 (0.3%)	0.7 (0.7)
		RE2. Having powerful images or memories that sometimes come into your mind in which you feel the experience is happening again in the here and now	951 (46.2%)	903 (43.9%)	142 (6.9%)	53 (2.6%)	10 (0.5%)	0.7 (0.8)
	Avoidance	AV1. Avoiding internal reminders of the experience (for example, thoughts, feelings, or physical sensations)	1,057 (51.3%)	738 (35.8%)	190 (9.2%)	63 (3.1%)	11 (0.5%)	0.7 (0.8)
		AV2. Avoiding external reminders of the experience (for example, people, places, conversations, objects, activities, or situations)	1,109 (53.9%)	736 (35.7%)	144 (7.0%)	57 (2.8%)	13 (0.6%)	0.6 (0.8)
	Sense of threat	TH1. Being “super-alert,” watchful, or on guard	1,129 (54.8%)	665 (32.3%)	176 (8.5%)	71 (3.4%)	18 (0.9%)	0.6 (0.8)
		TH2. Feeling jumpy or easily startled	1,053 (51.1%)	747 (36.3%)	182 (8.8%)	60 (2.9%)	17 (0.8%)	0.7 (0.8)
	PTSD functional impairment	PTSDI1. Affected your relationships or social life	1,430 (69.5%)	497 (24.1%)	96 (4.7%)	30 (1.5%)	6 (0.3%)	0.4 (0.7)
		PTSDI2. Affected your work or ability to work	1,431 (69.5%)	494 (24.0%)	95 (4.6%)	26 (1.3%)	13 (0.6%)	0.4 (0.7)
		PTSDI3. Affected any other important part of your life such as parenting, school or college work, or other important activities	1,402 (68.1%)	512 (24.9%)	94 (4.6%)	41 (2.0%)	10 (0.5%)	0.4 (0.7)
Disturbances in self-organization (DSO)	Affective Dysregulation	AD1. I react intensely to things that do not seem to affect other people so much	722 (35.1%)	971 (47.2%)	285 (13.8%)	68 (3.3%)	13 (0.6%)	0.87 (0.81)
		AD2. When I am upset, it takes me a long time to calm down	1,202 (58.4%)	636 (30.9%)	142 (6.9%)	66 (3.2%)	13 (0.6%)	0.57 (0.81)
	Negative self-concept	NSC1. I feel like a failure	985 (47.8%)	789 (38.3%)	185 (9.0%)	75 (3.6%)	25 (1.2%)	0.72 (0.86)
		NSC2. I feel worthless	1,207 (58.6%)	625 (30.4%)	145 (7.0%)	64 (3.1%)	18 (0.9%)	0.57 (0.82)
	Disturbances in relationships	DR1. I feel distant or cut off from people	1,290 (62.7%)	577 (28.0%)	127 (6.2%)	53 (2.6%)	12 (0.6%)	0.50 (0.77)
		DR2. I find it hard to stay emotionally close to people	1,142 (55.5%)	692 (33.6%)	147 (7.1%)	57 (2.8%)	21 (1.0%)	0.60 (0.82)
	DSO functional impairment	DSOI1. Affected your relationships or social life	1,213 (58.9%)	668 (32.4%)	127 (6.2%)	40 (1.9%)	11 (0.5%)	0.53 (0.74)
		DSOI2. Affected your work or ability to work	1,341 (65.1%)	571 (27.7%)	102 (5.0%)	37 (1.8%)	8 (0.4%)	0.45 (0.71)
		DSOI3. Affected any other important part of your life such as parenting, school or college work, or other important activities	1,355 (65.8%)	540 (26.2%)	114 (5.5%)	40 (1.9%)	10 (0.5%)	0.45 (0.73)
